# Supplementary material for: Tumor Accumulation of PIP-Based KRAS Inhibitor KR12 Evaluated by the Use of a Simple, Versatile Chicken Egg Tumor Model
Source: Cancers (Basel). 2022 Feb 14;14(4):951. doi: 10.3390/cancers14040951 (PMC8869854; doi:10.3390/cancers14040951)
Supplement: Supplementary file 1 [file cancers-14-00951-s001.zip › cancers-1576861-supplementary.pdf]

cancers

Supplementary Materials: Tumor Accumulation of PIP-Based KRAS Inhibitor KR12 Evaluated by the Use of a Simple, Versatile Chicken Egg Tumor Model

Yuya Higashi [1], Shuji Ikeda [2], Kotaro Matsumoto [1], Shinsuke Satoh [2], Aoi Komatsu [1], Hiroshi Sugiyama [2] and Fuyuhiko Tamanoi [1,*]

[1] Institute for Integrated Cell-Material Sciences, Institute for Advanced Study, Kyoto University, Kyoto 606-8501, Japan; higashi.yuya.5n@kyoto-u.ac.jp (Y.H.); matsumoto.kotaro.5r@kyoto-u.ac.jp (K.M.); komatsu.aoi.6z@kyoto-u.ac.jp (A.K.); sugiyama.hiroshi.3s@kyoto-u.ac.jp (H.S.)
[2] Department of Chemistry, Graduate School of Science, Kyoto University, Kyoto 606-8502, Japan; s-ik@kc5.so-net.ne.jp (S.I.); shinsuke.sato@elsevier.com (S.S.); sugiyama.hiroshi.3s@kyoto-u.ac.jp (H.S.)
* Correspondence: tamanoi.fuyuhiko.2c@kyoto-u.ac.jp; Tel.: +81-75-753-9856

1. Characterization of KR12-TAMRA by HPLC

Reversed-phase (RP) HPLC purification was performed on an Engineering PU-2089 plus series system (Jasco) using a COSMOSIL 5C18-MS-II column (Nacalai tesque). 0.1% TFA in water and acetonitrile were used as the eluent with detection at 254 nm. As shown in Figure S1, the sharp measure peak was detected. RP HPLC purification and lyophilization give KR12-TAMRA (4.6 mg, 1.9 µmol, 37 %) as a purple solid.

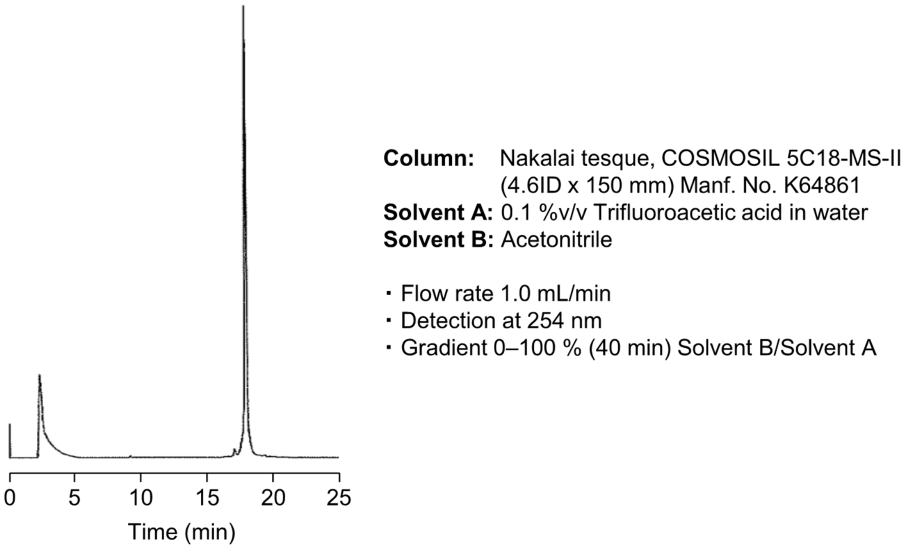

Figure S1. HPLC analysis of KR12-TAMRA.

2. Characterization of KR12-TAMRA by MALDI-TOF MS

Matrix assisted laser desorption/ionization time-of-flight mass spectrometry (MALDI-TOF MS) analysis was conducted on a microflex system (Bruker Daltonics K.K.). The purified KR12-TAMRA was characterized using MALDI-TOF MS. MALDI-TOF MS m/z calcd for C116H135N38O21+ [M + H]+ 2396.1, 2397.1, 2398.1, and found 2397.5, 2398.5, 2399.5 as shown in Figure S2.

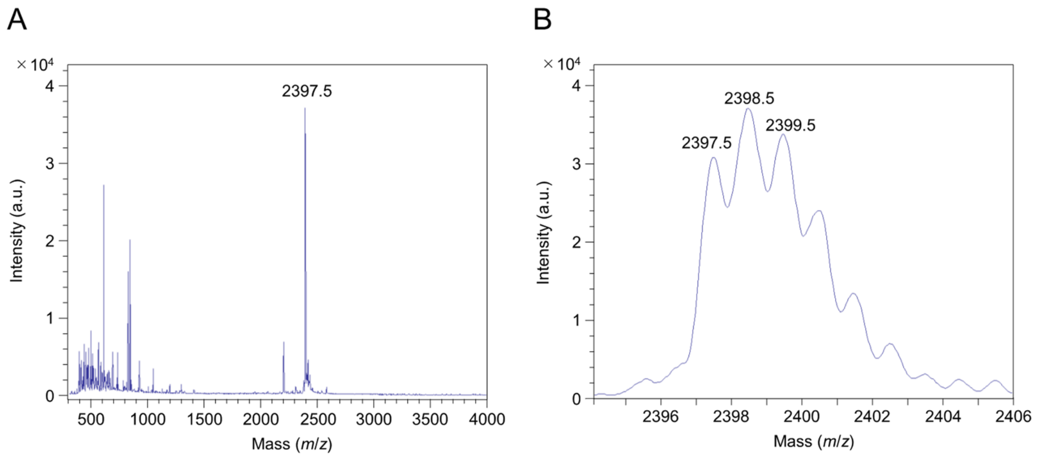

Figure S2. MALDI-TOF MS analysis of KR12-TAMRA. **A:** MALDI-TOF mass spectrum of KR12-TAMRA. **B:** Spectrum near the peak of KR12-TAMRA.
